# Condensin I Reveals New Insights on Mouse Meiotic Chromosome Structure and Dynamics

**DOI:** 10.1371/journal.pone.0000783

**Published:** 2007-08-22

**Authors:** Alberto Viera, Rocío Gómez, María T. Parra, John A. Schmiesing, Kyoko Yokomori, Julio S. Rufas, José A. Suja

**Affiliations:** 1 Departamento de Biología, Edificio de Biológicas, Universidad Autónoma de Madrid, Madrid, Spain; 2 Department of Biological Chemistry, College of Medicine, University of California at Irvine, Irvine, California, United States of America; Ordway Research Institute, United States of America

## Abstract

Chromosome shaping and individualization are necessary requisites to warrant the correct segregation of genomes in either mitotic or meiotic cell divisions. These processes are mainly prompted in vertebrates by three multiprotein complexes termed cohesin and condensin I and II. In the present study we have analyzed by immunostaining the appearance and subcellular distribution of condensin I in mouse mitotic and meiotic chromosomes. Our results demonstrate that in either mitotically or meiotically dividing cells, condensin I is loaded onto chromosomes by prometaphase. Condensin I is detectable as a fuzzy axial structure running inside chromatids of condensed chromosomes. The distribution of condensin I along the chromosome length is not uniform, since it preferentially accumulates close to the chromosome ends. Interestingly, these round accumulations found at the condensin I axes termini colocalized with telomere complexes. Additionally, we present the relative distribution of the condensin I and cohesin complexes in metaphase I bivalents. All these new data have allowed us to propose a comprehensive model for meiotic chromosome structure.

## Introduction

Chromosome compaction and condensation are indispensable requisites for the correct segregation of chromosomes during anaphase in mitosis and meiosis ([1]–[3]). In this sense, duplicated DNA molecules must progressively restructure and condense during prophase in order to individualize, package into rod-shaped structures, and resolve their chromatids prior to anaphase in order to prompt a proper chromosome segregation (for review see [4], [5]). Although the knowledge on this topic is continuously increasing, we are still far away from the complete understanding of the molecular machinery and the interrelated processes that lead to a correct chromosome compaction and condensation. In the past years, a multiprotein complex termed condensin was characterized in mitotic chromosomes from *Xenopus* egg extracts ([6], [7]). This condensin complex has revealed to be one of the main acting factors involved in chromosome condensation, maintenance of chromosome shaping, and proper sister chromatid segregation in anaphase from yeast to humans ([6]–[9]). In *Xenopus*, the condensin complex consists of five different subunits, SMC2 and SMC4, two members of the SMC (Structural Maintenance of Chromosomes) protein family, and three non-SMC proteins, CAP-G, CAP-D2 and the kleisin CAP-H ([1], [10]). Analogous complexes have been subsequently identified in a variety of species such as *Caenorhabditis elegans* ([11], [12]), *Schizosaccharomyces pombe* ([13]), *Saccharomyces cerevisiae* ([14]), or human ([15], [16]). It has been established that the hindrance of chromosomal condensation by either the blocking or depletion of any of the condensin subunits leads to segregation abnormalities and therefore cellular crisis in both mitotically ([2], [17]–[23]) and meiotically ([22]–[25]) dividing cells.

Recent investigations have revealed the existence of another holocomplex which shares the two SMC subunits in addition to the non-SMC subunits CAP-G2, CAP-D3, and the kleisin CAP-H2. This new complex has been termed condensin II ([26], [27]), and therefore the initially described complex has been renamed as condensin I. Both complexes possess different specific dynamic and functional characteristics (for review see [4], [5]). At least in vertebrate chromosomes, condensin I and II exhibit distinct patterns of distribution along the mitotic chromosome axis ([26], [28]), and whereas condensin II has been reported to locate to chromosomes as early as interphase, condensin I loads to chromosomes by early prometaphase ([28]). It has been recently proposed that condensin II may act as a primer organizer of chromatids along the axis, while condensin I would be responsible for the chromosomal final shaping ([26], [28]).

Despite all this background, little is known as regards to the appearance, location and dynamics of condensin in meiotic cells, being the initial studies reduced to *A. thaliana* ([23]), *C. elegans* ([22], [25]), *Xenopus* ([29]), and *S. cerevisiae* ([24]). Therefore, the aim of the present study was to determine the presence, loading, dynamics and participation of the condensin I complex in the structure of mammalian meiotic chromosomes. For this purpose we have analyzed by immunofluorescence the presence, subcellular distribution and cell cycle-regulated dynamics of condensin I in mitotic spermatogonia and spermatocytes during both meiotic divisions in male mouse. Moreover, we have double immunolabeled condensin I subunits with different telomere and centromere/kinetochores proteins in order to precisely determine the location of condensin I in condensed meiotic chromosomes. Additionally, we show for the first time in mammals the relative distribution of condensin and cohesin complexes in metaphase I chromosomes. According to our results we propose and discuss a comprehensive model for chromosome structure in meiosis.

## Results

### Immunoblotting

To test the specificity of the anti-hCAP-H antibody in mouse meiotic cells we performed an immunoblot analysis with extracts of mouse testis, using extracts of HeLa cultured cells as a control. The antibody specifically recognized a single protein band, in both HeLa and mouse testis extracts, that migrated at a same position representing approximately 90 kDa (Supplementary Fig. S1), which corresponds to the previously reported molecular weight of this protein in human cells ([30]). Thus, the anti-hCAP-H antibody also recognizes mouse CAP-H, indicating a high degree of conservation for this protein in mammals.

### Distribution of CAP-H during spermatogonial mitosis

We obtained almost an identical pattern of labeling for all the three non-SMC subunits of the condensin I complex during spermatogonial mitosis and both meiotic divisions. Thus, although we will only refer to those results obtained for CAP-H, identical patterns of distribution were observed for CAP-G, and CAP-D2, except where indicated, and are available as supplementary information.

**Figure 1:**
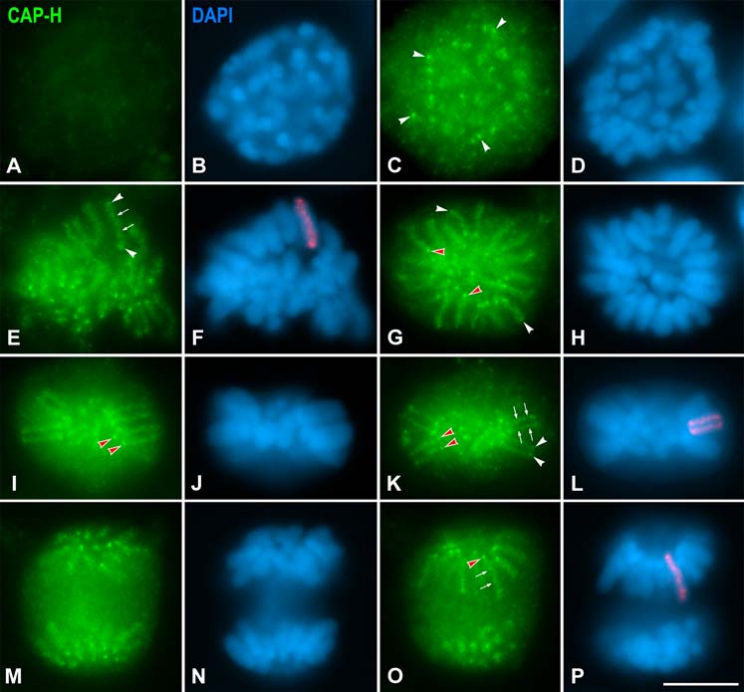
CAP-H distribution in spermatogonial mitosis. Mouse spermatogonia were stained for CAP-H (green) and counterstained with DAPI (blue). (A, B) CAP-H is not detected in early prophase. (C, D) In early prometaphase, condensing chromosomes show a faint and diffuse CAP-H labeling, but some bright accumulations (arrowheads) are observed on them. (E, F) In late prometaphase, CAP-H is detected as a single axis (arrows) running along and inside chromosomes. Note that the ends of these CAP-H axes (arrowheads) are brighter than the own axes. (G, H) Top view of a metaphase cell showing a ‘rosette’-like chromosome distribution. In these views a single CAP-H axis is seen in each chromosome. The centromeric (red arrowheads) and distal (white arrowheads) axes ends are brightly stained. (I–L) Lateral views of metaphase cells. In these views each chromosome shows two parallel CAP-H axes (arrows), one per sister chromatid. The centromeric (red arrowheads) and distal (white arrowheads) axes ends appear brightly stained. (M–P) Two different focal planes throughout an anaphase. A single CAP-H axis (arrows) is present inside each chromatid. In (F), (L) and (P) the CAP-H labeling on one chromosome/chromatid has been pseudocolored in red and superimposed on its corresponding DAPI image. Bar, 5 µm.

To study the distribution of the non-SMC condensin I subunits we employed the squash procedure since it preserves chromosome condensation and positioning in dividing spermatocytes, and also allows the unambiguous determination of all mitotic and meiotic stages ([31]). The distribution of CAP-H was first analyzed in mitotically proliferating spermatogonial cells present in squashed seminiferous tubules by using an anti-hCAP-H antibody. CAP-H was not detected in prophase nuclei (Fig. 1A and B), however, this antibody rendered a significant labeling on mitotic chromosomes from late prophase/early prometaphase up to telophase (Fig. 1C–P). In early prometaphase, a faint and diffuse CAP-H labeling was observed on the arms of condensing chromosomes, and bright and discrete CAP-H signals were additionally found at some chromosome regions (Fig. 1C and D). In late prometaphase chromosomes, a fuzzy CAP-H staining resembling a single coiled axial structure was detected inside them (arrows in Fig. 1E). Interestingly, the proximal and distal ends of those fuzzy CAP-H axes appeared as brighter spots (arrowheads in Fig. 1E). Since mouse chromosomes are telocentric ([32]), we will refer to proximal chromosome ends as those located at the centromere end, and to distal ones as those located at the non-centromeric end of the chromosome.

The observation of polar views of spermatogonial metaphases, displaying the characteristic “rosette”-like chromosome distribution (Fig. 1G and H), demonstrated that the chromosome distribution of CAP-H was identical to that observed in prometaphase. Each metaphase chromosome showed a single fuzzy CAP-H axis with two brighter dots at their ends, a proximal one (red arrowheads in Fig. 1G) close to the centromeric heterochromatin (discernible by a brighter DAPI staining; Fig. 1H), and a distal one (white arrowheads in Fig. 1G) near the non-centromeric end of the chromosome. Nevertheless, the observation of lateral views of spermatogonial metaphases (Fig. 1I–L) revealed one CAP-H axis (arrows in Fig. 1K) inside each sister chromatid (Fig. 1L). These sister axes appeared enlarged at both their proximal (red arrowheads in Fig. 1I and K) and distal (white arrowheads in Fig. 1K) ends, although the proximal dots were brighter than the distal ones. This CAP-H labeling was also observed in segregating chromatids during anaphase (Fig. 1M–P). CAP-G (data not shown) and CAP-D2 (Supplementary Fig. S2) showed the same pattern of chromosome distribution as CAP-H during spermatogonial mitosis. Therefore, the distribution of condensin I subunits in mouse spermatogonial mitotic chromosomes is similar to that previously reported for human mitotic chromosomes ([26]), except for the preferential accumulations at chromosome ends.

The phosphorylation of histone H3 at serine 10 (pH3) is considered the earliest event defining the onset of mitotic condensation ([33]). In order to establish the timing of association of CAP-H relative to the initiation of chromosome condensation, we made a double immunolabeling of CAP-H and pH3. Our results showed that CAP-H was recruited to chromosomes after the phosphorylation of histone H3 (Supplementary Fig. S3).

### Distribution of CAP-H during meiosis I

**Figure 2:**
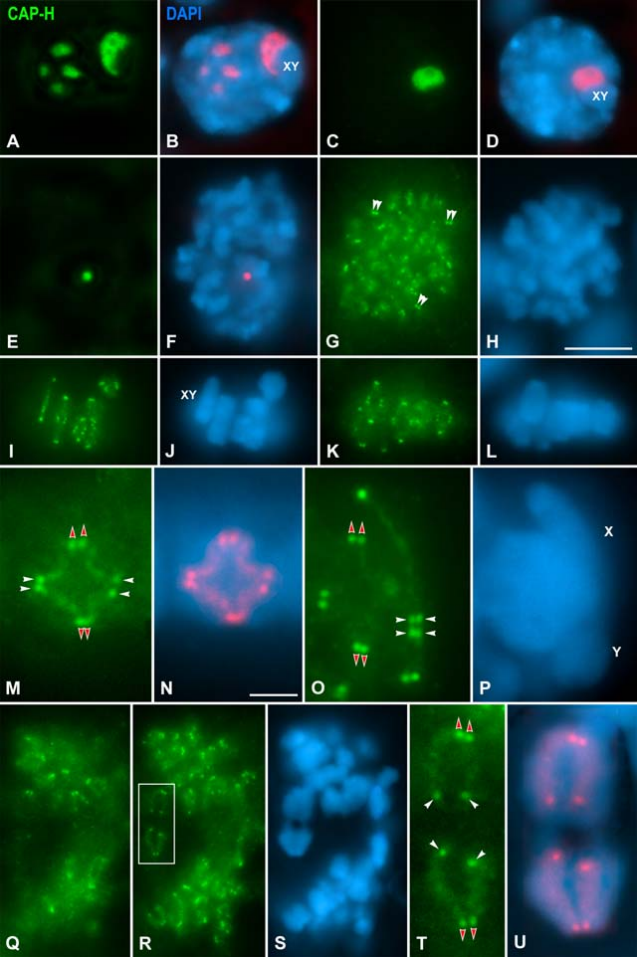
CAP-H distribution in meiosis I spermatocytes. Mouse spermatocytes were stained for CAP-H (green) and counterstained with DAPI (blue). (A, B) In pachytene spermatocytes, CAP-H is detected in nucleoli lying in the nucleoplasm and associated to the sex body (XY). (C, D) Diplotene spermatocytes exhibit a CAP-H accumulation in the nucleolus associated to the sex body (XY). (E, F) In diakinesis, CAP-H is detected in a nucleolar remnant in the nucleoplasm. (G, H) In prometaphase I spermatocytes, a faint CAP-H labeling is observed along bivalents, and pairs of bright dots (double arrowheads) at chromosome ends. (I–L) Two focal planes throughout a metaphase I spermatocyte. The autosomal and sex (XY) bivalents show pairs of bright CAP-H spots at their centromeric and distal ends. (M–P) Selected autosomal and sex (XY) metaphase I bivalents. Four pairs of bright CAP-H spots are detected in each bivalent, one pair at each centromeric chromosome end (red arrowheads), and one pair at each distal chromosome end (white arrowheads). In (M) a diffuse CAP-H axis is observed inside each chromatid between the proximal and distal spots. (Q-S) Two focal planes of an anaphase I spermatocyte. (T, U) Segregating half-bivalents boxed in (R). In each chromosome, a pair of CAP-H dots is detected at the centromere region (red arrowheads), one spot at the distal end of each chromatid (white arrowheads), and a diffuse axial labeling along chromatids. In (B), (D), (F), (N) and (U) the CAP-H staining has been pseudocolored in red and superimposed on its corresponding DAPI image. Bars: (A–L, Q–S) 5 µm; (M–P, T and U) 3 µm.

During meiosis I, CAP-H was first detected in pachytene spermatocytes. In these spermatocytes, several large CAP-H accumulations, about five, were observed scattered in the nucleoplasm (Fig. 2A and B). One of these CAP-H accumulations was always associated to the sex body (Fig. 2A and B). From late pachytene up to diplotene, the number of nucleoplasmic CAP-H accumulations decreased until only a single CAP-H accumulation remained visible at the sex body periphery (Fig. 2C and D). In diakinesis spermatocytes, a single small and round CAP-H accumulation was visible in the nucleoplasm (Fig. 2E and F). The dynamics of the CAP-H accumulations from pachytene up to diakinesis strongly resembled that of nucleoli in prophase I mouse spermatocytes ([34]). The double immunolabeling of CAP-H and fibrillarin, a nucleolar protein mainly located at the dense fibrillar component ([35]), demonstrated that CAP-H was present at nucleoli from pachytene up to diakinesis (Supplementary Fig. S4). It is worth mentioning that whereas CAP-H concentrated at nucleoli in prophase I spermatocytes, CAP-D2 and CAP-G were only detected on chromosomes from prometaphase I on (data not shown). In these spermatocytes, CAP-H, as CAP-D2 (Supplementary Fig. S5) and CAP-G, were detected as a faint labeling on bivalents (Fig. 2G and H), and as pairs of bright dots (double arrowheads in Fig. 2G). During metaphase I, the sex and the autosomal bivalents (Fig. 2I–P), presented fuzzy CAP-H axial structures inside their chromatids, and brightly labeled pairs of dots at their proximal and distal ends (Fig. 2I–L). In favorable bivalents, the presence of four diffuse CAP-H axes was discerned along the inner region of each chromatid between the proximal and distal spots (Fig. 2M and N). As a rule, four pairs of bright CAP-H spots were clearly discerned in each bivalent, even in the sex one (Fig. 2M–P), a pair close to each homologous centromere (red arrowheads in Fig. 2M and O), and another one near each distal chromosome end (white arrowheads in Fig. 2M and O). Segregating anaphase I half-bivalents displayed diffuse CAP-H axes, and brighter spots at their ends (Fig. 2Q–S). Within a given chromosome, a pair of closely related CAP-H dots was distinguished at the centromeric ends of the axes, and a single spot, located at the other axis end, near the distal tip of each chromatid (Fig. 2T and U).

A double immunolabeling of CAP-H and pH3 on meiosis I spermatocytes showed that pH3 was first detected at the heterochromatic chromocentres in diplotene spermatocytes as previously reported ([36]), while CAP-H was still concentrated at nucleoli (Supplementary Fig. S6).

### Distribution of CAP-H during meiosis II

**Figure 3:**
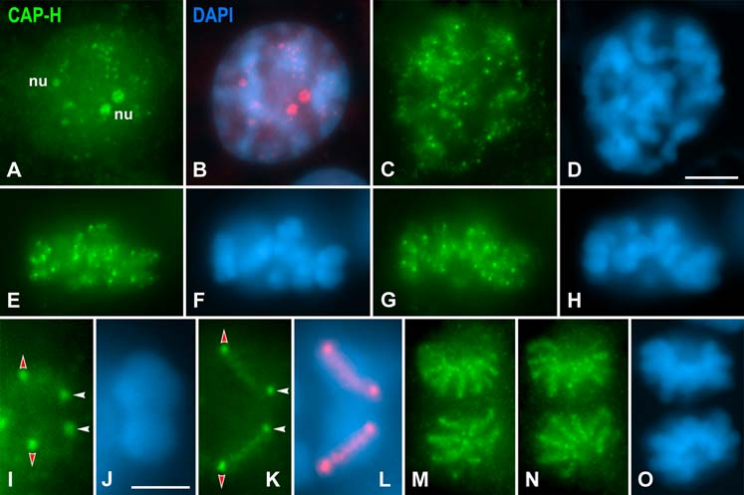
CAP-H distribution in meiosis II spermatocytes. Mouse spermatocytes were stained for CAP-H (green) and counterstained with DAPI (blue). (A, B) Late interkinesis nucleus. CAP-H appears at several nucleoli (nu) and as small spots in the nucleoplasm. (C, D) Prophase II spermatocyte. A diffuse CAP-H labeling, and bright spots, are observed along chromosomes. (E–H) Two different focal planes of a metaphase II spermatocyte. Bright CAP-H spots are present at chromosome ends. (I, J) Selected metaphase II chromosome. Each chromatid shows two CAP-H spots, one at the centromeric end (red arrowheads) and one at its distal end (white arrowhead). (K, L) Selected segregating chromatids in early anaphase II. Both chromatids present a CAP-H axis and one spot at each axis end. (M–O) Two focal planes of a late anaphase II spermatocyte. Each chromatid exhibits a single diffuse CAP-H axis between the centromeric and distal ends spots. In (B) and (L) the CAP-H labeling has been pseudocolored in red and superimposed on its corresponding DAPI image. Bars: (A–H, M–O) 5 µm; (I–L) 3 µm.

In late interkinesis nuclei, CAP-H appeared as nucleoplasmic accumulations representing nucleoli, and as small spots (Fig. 3A and B). Afterwards, in prophase II, condensing chromosomes presented a diffuse CAP-H labeling and bright spots close to their ends (Fig. 3C and D). Metaphase II chromosomes displayed a blurred CAP-H staining inside their chromatids and four brighter spots near their ends (Fig. 3E–H). Two separated proximal CAP-H spots were positioned at the centromere region (red arrowheads in Fig. 3I), and two additional ones at the distal end of each chromatid (white arrowheads in Fig. 3I). At the onset of anaphase II (Fig. 3K and L), segregating chromatids presented a central CAP-H axis and one spot at either the proximal (red arrowheads in Fig. 3K), and distal (white arrowheads in Fig. 3K) axis ends. This distribution of CAP-H was similar in anaphase II spermatocytes (Fig. 3M–O).

A double immunolabeling of CAP-H and pH3 on meiosis II spermatocytes showed that CAP-H was detected at nucleoli and small spots in late interkinesis nuclei, when their chromatin was completely labeled by pH3 (Supplementary Fig. S7).

### CAP-H preferentially localizes to telomere complexes

In order to accurately determine the localization of CAP-H at the ends of condensed meiotic chromosomes, we performed double immunolabeling experiments using either an anti-centromere ACA serum, revealing kinetochores, or an anti-TRF1 antibody to detect the telomere complexes.

**Figure 4:**
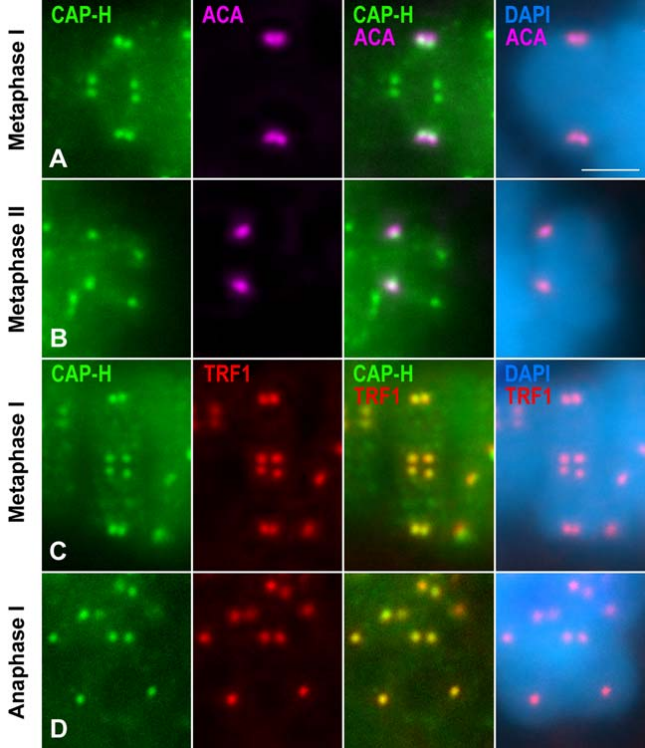
CAP-H accumulates preferentially at telomere complexes. Selected bivalents and chromosomes were stained for CAP-H (green), kinetochores revealed by an ACA serum (pink), TRF1 (red), and counterstained with DAPI (blue). (A) Metaphase I bivalent. A pair of CAP-H dots at the centromere region of each chromosome is below the closely associated sister kinetochores. (B) Metaphase II chromosome. The separated CAP-H spots at the centromere region appear below the kinetochores. (C) Metaphase I bivalent and (D) anaphase I half-bivalent. The CAP-H dots colocalize with the TRF1 signals. Bar, 3 µm.

The ACA serum revealed a pair of closely associated signals, representing sister kinetochores, at each homologous centromere in metaphase I bivalents (Fig. 4A). The pair of centromeric CAP-H spots was smaller, and appeared beneath the closely associated sister kinetochores (Fig. 4A). This relative distribution was maintained at centromere regions of anaphase I half-bivalents (data not shown). In metaphase II chromosomes, sister kinetochores appeared as two round individualized signals facing opposite poles (Fig. 4B). One proximal CAP-H signal appeared below each kinetochore (Fig. 4B). Therefore, the proximal accumulations of condensin I were located beneath the kinetochores in condensed chromosomes during both meiotic divisions.

Since the distribution of the bright accumulations of CAP-H at the axes ends, from prometaphase I up to telophase II, presented a similar distribution to that previously described for the telomere complexes in condensed mouse meiotic chromosomes ([37]), we performed a double immunolabeling of CAP-H and TRF1. When the chromosomal distribution of CAP-H was analyzed in comparison to that of TRF1, it became evident that not only their distribution, but their shape and size were similar. In this sense, in a given metaphase I autosomal bivalent, the eight bright CAP-H accumulations detected near the ends of the chromosomes, colocalized with TRF1 at both the proximal and distal telomere complexes (Fig. 4C). Likewise, in anaphase I half-bivalents, the pair of closely related CAP-H accumulations near the centromere, and the CAP-H accumulations at the non-centromeric end of each chromatid colocalized with the corresponding TRF1-labeled telomere complexes (Fig. 4D). It is worth noting that this preferential localization of CAP-H onto telomere complexes was maintained throughout meiosis II until telophase II (data not shown). Moreover, the accumulations of CAP-H at the ends of the chromatid axes also colocalized with TRF1 in spermatogonial metaphase chromosomes (Supplementary Fig. S8). Therefore, all these observations strongly support that condensin I complexes are preferentially recruited to the telomere complexes of condensed chromosomes during male mouse meiosis and mitosis.

### Relative distribution of condensin I and RAD21 cohesin complexes in meiosis I chromosomes

In order to precisely determine the relative distribution of condensin I and cohesin complexes in mouse meiotic chromosomes we performed a double immunolabeling of CAP-H and the cohesin subunit RAD21, that is expressed during male mouse meiosis ([38]).

**Figure 5:**
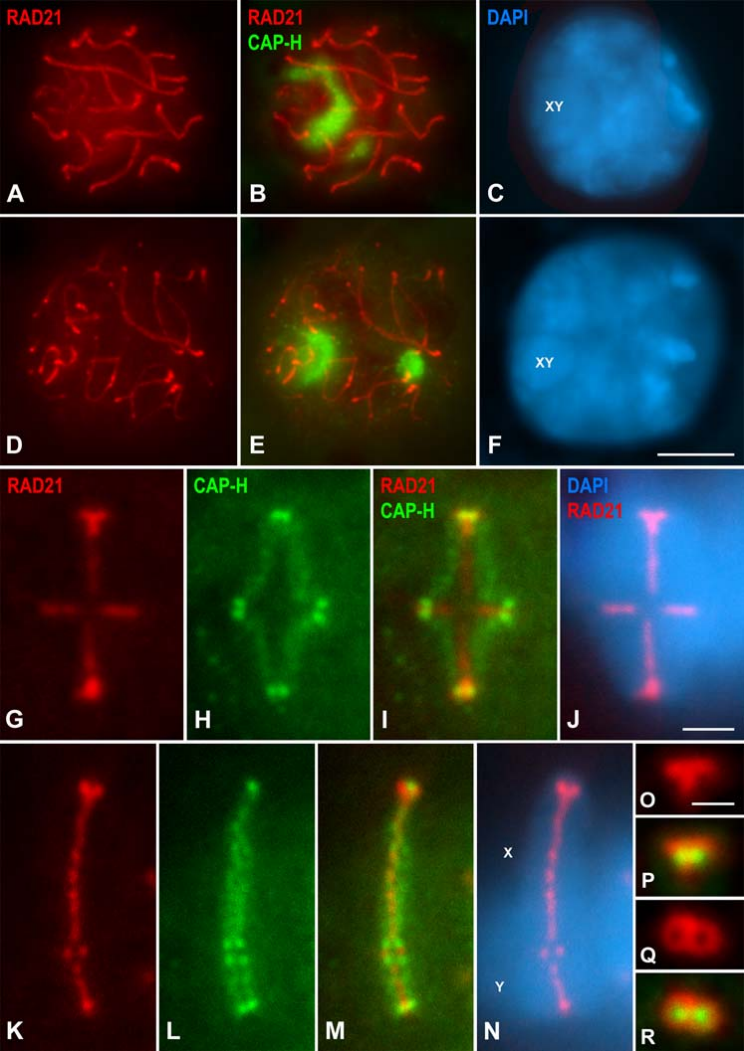
Distribution of CAP-H and the cohesin subunit RAD21 in meiotic chromosomes. Mouse spermatocytes were stained for RAD21 (red), CAP-H (green) and counterstained with DAPI (blue). (A-C) Pachytene spermatocyte. RAD21 is located on the autosomal lateral elements and unsynapsed sex (XY) axial elements, while CAP-H concentrates at nucleoli. (D-F) Diplotene. RAD21 appears on either the desynapsed autosomal lateral elements, or the unsynapsed sex (XY) axial elements, and CAP-H remains associated to nucleoli. (G-J) Selected metaphase I autosomal bivalent. RAD21 appears enriched at homologous centromeres, conforming a T-shaped structure, and as a series of fainter patches at the interchromatid domain. Note that the RAD21 labeling is interrupted at the interstitial chiasma. By contrast, the CAP-H labeling is found as diffuse axes along the inner region of each chromatid, and as brighter spots at the centromeric and distal axes ends. (K-N) Selected metaphase I sex bivalent. The labeling patterns of RAD21 and CAP-H in sex bivalents resemble those found on autosomes, except for a larger and more continuous RAD21 labeling at the interchromatid domain. (O, P) Enlarged side view of a metaphase I centromere. RAD21 appears as a T-shaped structure, and the CAP-H spots partially colocalize with the middle region of the RAD21 signal. (Q, R) Enlarged top view of a metaphase I centromere. RAD21 appears as two associated rings that surround the CAP-H spots. Bars: (A–F) 5 µm; (G–N) 3 µm; (O–R) 1.5 µm.

Pachytene spermatocytes evidenced RAD21 signals along cohesin axes that are coincident with the fully synapsed autosomal lateral elements (LEs) of the synaptonemal complex (SC), and the unsynapsed sex axial elements (AEs) (Fig. 5A-C). By contrast, CAP-H appeared at nucleoli lying in the nucleoplasm and at the sex body periphery (Fig. 5B). In diplotene spermatocytes, RAD21 was detected along desynapsing autosomal LEs and at unsynapsed sex AEs (Fig. 5D-F). In this stage, CAP-H remained located at nucleoli (Fig. 5E). Afterwards, from diakinesis up to prometaphase I, RAD21 faded away from desynapsed LEs, and CAP-H disappeared from nucleoli.

In either the autosomal or sex metaphase I bivalents, RAD21 was observed at the so-called interchromatid domain, and at centromeres (Fig. 5G–N). An accurate analysis of autosomal metaphase I bivalents revealed a series of small RAD21 patches at the interchromatid domain, from centromeres towards distal telomeres (Fig. 5G). Interestingly, this labeling was usually brighter and more continuous in the sex bivalent (Fig 5K). This interchromatid domain RAD21 labeling was interrupted at chiasma sites in both autosomal and sex metaphase I bivalents (Fig. 5G and K). Despite the localization at the interchromatid domain, RAD21 appeared concentrated onto T-shaped structures at the centromeres of all autosomes (Fig. 5G) and the centromere of the X chromosome (Fig. 5K). By contrast, the CAP-H labeling, as previously described, was found as a fuzzy axial structure at the inner region of each chromatid, with brighter accumulations at their proximal and distal ends (Fig. 5H and L). The merged images of RAD21 and CAP-H in metaphase I bivalents showed that whereas RAD21 was located at the interchromatid domain between the sister chromatids, CAP-H appeared at their inner regions (Fig. 5I and M). A detailed examination of side-viewed metaphase I centromeres showed that the proximal CAP-H accumulations flanked the RAD21 T-shaped structure (Fig. 5O and P). Interestingly, centromere top views demonstrated the presence of two side-by-side associated RAD21 rings (Fig. 5Q), with the proximal CAP-H spots located slightly beneath their cavities (Fig. 5R). Consequently, proximal CAP-H spots, which partially colocalized with the RAD21 T-like structure, appear at the so-called inner centromere domain of metaphase I chromosomes ([36]), as previously described for proximal telomere complexes in mouse condensed meiotic chromosomes ([37]).

## Discussion

### Recruitment of condensin I to spermatogonial mitotic chromosomes

We have found that the non-SMC subunits of the condensin I complex CAP-G, CAP-D2, and CAP-H, become associated to chromosomes in mouse spermatogonial cells during prometaphase. Consistently, our results show that these subunits are recruited after the phosphorylation of histone H3 at serine 10, a landmark of the initiation of mitotic condensation ([33]). Thus, our data support those obtained in mammalian and plant somatic cells reporting the association of condensin I complexes to chromosomes only after the nuclear envelope breakdown in prometaphase ([9], [28], [30], [39], [40]). Therefore, our observations revealed an identical loading pattern of condensin I in somatic and male germ cells. This contrasts with condensin II complexes, which are loaded to chromosomes during prophase ([28], [39], [40]).

### Nucleolar localization of condensin I

In striking contrast with our observations in spermatogonial cells, CAP-H was the only non-SMC subunit of condensin I detectable inside prophase spermatocyte nuclei during both meiotic divisions. CAP-H localized at nucleoli from pachytene up to diakinesis during prophase I, and also in late interkinesis and prophase II nuclei. This nucleolar localization of CAP-H, and also of CAP-C and CAP-E, has been previously described in interphase human HeLa cells ([30]). Likewise, some condensin I and II subunits have been found at nucleoli in interphase *Xenopus* ([41]), *Arabidopsis* and tobacco cultured cells ([40]), and also in *Xenopus* oocytes ([42]). It has been suggested that in higher eukaryotes the condensin complex may participate in the chromatin remodeling and condensation-dependent silencing of rDNA loci ([30]). Alternatively, it has been suggested that condensin may alter the secondary structure of rRNAs or play a scaffolding role in spatial organization of nucleoli ([41]), or may be sequestered at nucleoli to facilitate chromosome decondensation during telophase ([30]). However, the putative nucleolar function of condensin complexes, or even condensin subunits, during mitosis and meiosis remains to be explored.

### Recruitment of condensin I to meiotic chromosomes

Our results indicate that condensin I appears at chromatid axes by prometaphase during both meiotic divisions and spermatogonial mitosis. Thus, our results are similar to those previously reported during *C. elegans* meiosis showing that MIX-1, a SMC2 homologue, and HCP-6, a homologue of the condensin II subunit CAP-D3, are recruited to chromosomes by late prophase I, during diakinesis ([25]). The condensin I subunits whose distribution we have analyzed are located at the inner region of the condensed chromatids during both meiotic divisions, as occurs in mitotic *Xenopus*, mammalian, plant, chicken and *Drosophila* chromosomes ([6], [8], [9], [15], [20], [26], [28], [39], [40]). Altogether, these results suggest that condensin I complexes are recruited to the chromatid axes of prometaphase chromosomes not only during mitosis, but also during both meiotic divisions.

### Telomeric accumulation of condensin I

Our results show that condensin I complexes are particularly enriched at the ends of the chromatid axes in mouse mitotic and meiotic chromosomes. Double immunolabelings with an anti-centromere autoantibody revealing kinetochores and TRF1, a telomeric protein, demonstrate that the proximal condensin I accumulations are located below the kinetochores. Moreover, the proximal and distal accumulations of condensin I colocalized with the proximal and distal telomere complexes, respectively ([37]). As far as we know, this is the first time that condensin I complexes are shown to be preferentially accumulated at telomere complexes.

The localization of proximal telomere and condensin I complexes below kinetochores is intriguing at first sight. All the chromosomes from the C57BL/6 mouse strain, except the Y chromosome, are telocentric since a short arm is not detected at a cytogenetic level. Thus, the proximal telomere and condensin I complexes observed below kinetochores do not correspond to a short arm. Previously, we have reported that in pachytene and diplotene mouse chromosomes the proximal telomeric DNA repeats and specific telomeric proteins are associated to the nuclear envelope, and that centromeres are internally located relative to the telomere complexes ([37]). By contrast, from metaphase I on, the proximal telomere complexes are located below the kinetochores. These results led us to suggest that in mouse meiotic chromosomes, there is a relocation of the proximal telomere complexes and kinetochores from diakinesis up to metaphase I, once the telomeres have detached from the nuclear envelope, in order to protect the proximal telomeric repeats and allow the interaction of microtubules with the kinetochores ([37]). In this sense, the reorganization of the proximal telomere complexes and kinetochores would be possible since it has been reported that the proximal telomere repeats and the centromeric minor satellite DNA repeats, that organize the kinetochores, are relatively separated by other repetitive sequences ([32], [43], [44]).

The preferential accumulation of condensin I complexes at telomere complexes in mouse mitotic and meiotic chromosomes might be due to the ultra-long arrays of telomeric DNA repeats found in mouse chromosomes ([45]–[47]). In this respect, established mouse strains, such as C57BL/6, have long hypervariable telomeric DNA lengths if compared with strains recently derived from wild mice ([48]). Most studies analyzing the distribution of telomeric DNA repeats in mammalian chromosomes by FISH have revealed that these repeats do not appear as large chromatin loops, as expected according to the scaffold/radial loop model of chromosome structure ([49]). By contrast, the telomeric repeats are detected at the cytological telomeres as discrete spots surrounded by chromatin in both mitotic ([50], [51]) and meiotic chromosomes ([52]–[56]). Our results suggest that the large accumulations of condensin I at telomere complexes may contribute to the specific organization of telomeric DNA in condensed mouse chromosomes. In this sense, the high number of telomeric repeats found in mouse could be organized as short loops in condensed chromosomes, as in prophase I chromosomes ([53]), that are maintained in a condensed state by means of their interaction with condensin I complexes. Since condensin I is located at chromatid axes along the arms, i.e. at the putative bases of the chromatin loops, the accumulation of condensin I at telomere complexes indicates that the number of loop bases is higher at telomeres than along the arms. Consequently, the presence of a high number of short telomeric DNA loops would explain the accumulation of condensin I complexes at their bases in mouse chromosomes. Alternatively, the telomeric DNA repeats are not organized as loops, and the condensin I complexes would allow their compaction in an undetermined organization. In our opinion these two alternatives are not mutually exclusive and should be tested in the future.

Our results show that in mouse chromosomes the proximal condensin I complexes do not colocalize with kinetochores. This is consistent with results obtained in human chromosomes where condensin I complexes are not present at kinetochores. However, condensin II complexes are enriched near the inner kinetochore plates, and depletion of condensin II subunits promotes defects in kinetochore structure and function ([28]). Consequently, further studies are needed to know whether condensin II complexes are also needed to organize kinetochores in mouse mitotic and meiotic chromosomes.

### Change of chromosome structure during meiosis I

**Figure 6:**
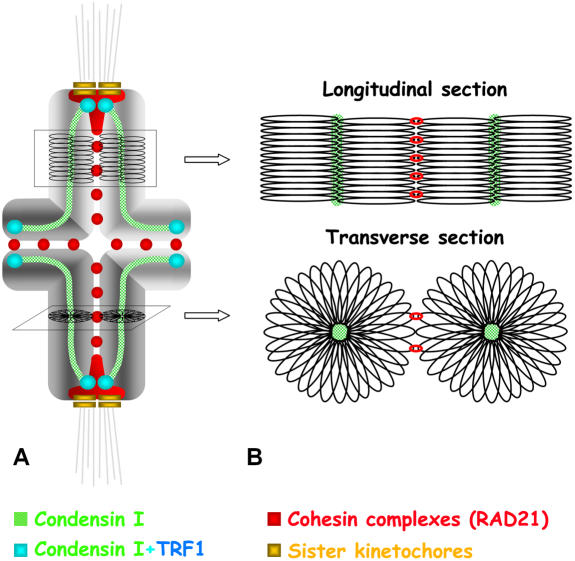
Representation of a metaphase I bivalent showing the relative distributions of condensin I and RAD21-containing cohesin complexes. One chromosome is depicted in light grey and its homologue is darker grey. Chromosomes are telocentric, and the metaphase I bivalent shows a single interstitial chiasma. Kinetochores are indicated in yellow, kMTs in light grey, condensin I in green, RAD21 in red, and TRF1 in blue. The colocalisation of condensin I and TRF1 is shown in light blue. In (A), the condensin I complexes delineate a fuzzy axis inside each chromatid, with prominent accumulations at their proximal and distal ends which colocalize with TRF1. The two proximal condensin I accumulations appear below the closely associated sister kinetochores, and partially colocalize with the middle region of the T-shaped RAD21 structure at each centromere. RAD21-containing cohesin complexes are also depicted as patches at the interchromatid domain. (B) Hypothetical model accounting for the distribution of condensin I and RAD21-containing cohesin complexes in relation to radial chromatin loops in a mouse metaphase I bivalent. The longitudinal and transverse sections of the arms correspond to areas indicated in (A).

There are no data concerning the appearance and location of the chromatid axes in condensed mammalian meiotic chromosomes (for review see [57]). We have found that condensin I axes, as well as silver-stained chromatid axes (Supplementary Fig. S9), are located at the inner region of each metaphase I chromatid. Consequently, and assuming the scaffold/radial loop model of chromosome structure, mouse metaphase I chromosomes show an organization similar to that found in mitotic metaphase chromosomes ([4], [57]) (Fig. 6). This situation is similar to that found in grasshopper metaphase I chromosomes. In these species, silver-stained chromatid axes are peripherally located with respect to the width of each chromatid by early metaphase I, so that sister axes are close to each other ([58]–[60]). However, the sister axes individualize all along their length to appear at the inner region of each chromatid by late metaphase I ([61]–[63]).

The appearance of chromatid axes at the inner region of metaphase I chromatids contrasts with their distribution in prophase I pachytene chromosomes. In rooster pachytene chromosomes, topo IIα, a component of the chromatid axes of mitotic chromosomes together with condensin I and II complexes, appears located on the outer edge of the synaptonemal complex LEs ([64]). Similarly, condensin complexes are present at pachytene LEs during budding yeast meiosis ([24]). These results indicate that the chromatid axes from both sister chromatids are intimately associated at the LE. Since, chromatin is organized as loops whose bases attach to LEs, and LEs are peripherally located in relation to the chromatin loops from both sister chromatids, it is said that pachytene chromosomes show a ‘meiotic organization’ (for review see [57]). Thus, chromosome organization must change from a meiotic one during pachytene to a mitotic one in metaphase I. While in grasshoppers this change of chromosome organization occurs throughout metaphase I, in male mouse this must happen between diplotene and metaphase I. Thus, during meiosis, different species change their chromosome structure at different times. In this sense, what seems to be really crucial is that chromosomes must show a mitotic organization previous to the release of sister-chromatid arm cohesion at the onset of anaphase I.

### Redistribution of cohesin complexes during meiosis I

The different cohesin complexes found during mammalian meiosis are located at cohesin axes coincident with the LEs during prophase I (for review see [65]). Thus, cohesin complexes are located at or near the bases of the chromatin loops from both sister chromatids (for review see [57]). Our observations demonstrate that cohesin complexes are distributed at the interchromatid domain in metaphase I bivalents, and not embracing both sister chromatids. Consequently, and according to the scaffold/radial loop model, the cohesin complexes would embrace the distal end of chromatin loops from both sister chromatids (Fig. 6). These observations suggest that, concomitantly with the remodeling of chromosome structure from a meiotic to a mitotic organization, i.e. from pachytene to metaphase I, cohesin complexes are redistributed from the bases of the chromatin loops to their distal ends. In this sense, it has been recently demonstrated in budding yeast that there is a condensin-dependent removal of a subset of cohesin complexes during prophase I that is dependent on the phosphorylation of the cohesin subunit Rec8 by Cdc5, a Polo-like kinase ([66]). A similar Polo-like kinase-dependent pathway to remove most cohesin complexes from chromosome arms also occurs during prophase in mitotic chromosomes ([67]-[69]). Likewise, it has been previously shown that there is a removal of a subset of cohesin complexes from chromosome arms during late diplotene and diakinesis in male mouse meiosis ([38], [70]). Since SGO2, a protector of centromeric cohesin complexes, appears only at centromeres during late diplotene and diakinesis in mouse spermatocytes ([71]), it is tempting to suggest that SGO2 would not protect arm cohesin complexes against their removal by a putative phosphorylation-dependent pathway during late prophase I stages. Accordingly, we propose that during diplotene and diakinesis, a partial loss of cohesin complexes from the arms could facilitate the resolution of sister chromatids, concomitantly with a change of chromosome structure from a meiotic to a mitotic organization. In this scenario, the cohesin complexes remaining at the arms during diakinesis might redistribute, for instance by a sliding mechanism, from the bases of chromatin loops to their ends in order to ensure sister-chromatid arm cohesion until the onset of anaphase I. Alternatively, some cohesin complexes might be removed from the arms during diakinesis and reloaded during or after chromosome structure remodelling. Obviously, further studies are needed to verify these hypotheses.

## Materials and Methods

### Cell culture

HeLa cultured cells were grown in flasks in Dulbecco's modified Eagle's medium (DMEM) containing 10% fetal calf serum, 50 units/ml penicillin, 50 µg/ml streptomycin and 1% 0.2 M L-glutamine (all from Imperial Laboratories, Andover, United Kingdom), at 37°C in a humidified atmosphere containing 5% CO_2_.

### Electrophoresis and immunoblotting

HeLa cells extracts were prepared by harvesting cultured cells, which were washed twice with PBS (137 mM NaCl, 2.7 mM KCl, 10.1 mM Na_2_HPO_4_, 1.7 mM KH_2_PO_4_, [pH 7.4]). Then, they were lysed in boiling SDS sample buffer (50 mM Tris-ClH pH 6.8, 3% SDS, 2 mM EDTA, 15% sucrose, 9% β-mercaptoethanol, 0.005% bromophenol blue).

Testis from adult male C57BL/6 mice were removed and placed in 2 ml of SDS solubilization solution (50 mM Tris-ClH pH 6.8, 5 mM EDTA, 3% SDS, 1% protease inhibitor cocktail). Then, testes were homogenized on ice in a Potter homogenizer. The extract was placed in boiling water bath for 5 minutes. The appropriate quantity of extract was diluted with 5X SDS-lysis buffer (62.5 mM Tris-ClH pH 6.8, 2% SDS, 5% β-mercaptoethanol, 10% glycerol, 0.005% bromophenol blue) and boiled for 5 minutes.

SDS-PAGE was carried out in 10% polyacrylamide gels. Gels were electrically transferred to Trans-Blot sheets (Bio-Rad, Hercules, California, United States) for 1.5 hour at 4°C and 310 mA. Sheets were blocked for 1 hour with 4% non-fat dry milk in PBS, followed by an overnight incubation at 4°C with an anti-hCAP-H antibody at a 1:10,000 dilution in 4% non-fat dry milk in PBS. Immunoreactive bands were visualized by incubation for 1 hour at room temperature with horseradish peroxidase-conjugated donkey anti-rabbit Ig (Amersham Life Science, Little Chalfont, United Kingdom) at a 1:5,000 dilution in PBS, and subsequent development using an ECL (Enhanced Chemiluminescence) detection system (Amersham Life Science) according to the manufacturer instructions.

### Squashing of spermatocytes

Adult male C57BL/6 mice were used in the present study. Testis were removed, detunicated, and seminiferous tubules then processed for squashing. For squashing we followed the technique previously described by Parra et al. ([31]). Briefly, seminiferous tubules were fixed in freshly prepared 2% formaldehyde in PBS containing 0.1% Triton X-100. After 5 minutes several seminiferous tubules fragments were placed on a clean slide coated with 1 mg/ml poly-L-lysine (Sigma, St. Louis, Missouri, United States) with a small drop of fixative, and the tubules were gently minced with tweezers. The tubules were then squashed and the coverslip removed after freezing in liquid nitrogen.

### Immunofluorescence microscopy

After fixation, the slides were rinsed three times for 5 minutes in PBS, and incubated for 45 minutes at room temperature with primary antibodies. Several subunits of the condensin I complex were detected. CAP-H was detected with a rabbit polyclonal antibody generated against a 211 aa amino-terminal fragment of human CAP-H ([72]), at a 1:100 dilution in PBS. To detect CAP-G a polyclonal rabbit serum raised against a recombinant polypeptide from the middle region (amino acids 235 to 570) of human CAP-G ([73]) was employed at a 1:100 dilution in PBS. Two polyclonal rabbit antisera which respectively recognize the amino-terminal (amino acids 997 to 1401) and the carboxyl-terminal (amino acids 1 to 241) subdomains of human hCAP-D2 (CNAP1) ([15]) were employed to detect CAP-D2 at a 1:20 dilution in PBS. Kinetochores were detected with the human autoimmune anti-centromere (ACA) serum GS, that recognizes CENP-A, -B and -C (kindly provided by Dr. William Earnshaw; [74]), at a 1:10,000 dilution in PBS. To detect the telomeric protein TRF1 we used a polyclonal rabbit serum (#TRF12-S; Alpha Diagnostic International, San Antonio, Texas, United States), raised against mouse TRF1, at a 1:100 dilution in PBS ([37]). To detect the cohesin subunit RAD21 we used the polyclonal rabbit serum K854 (kindly provided by Dr. José Luis Barbero; [75]), at a 1:50 dilution in PBS. Fibrillarin was detected with the S4 human anti-fibrillarin autoantibody at a 1:500 dilution (kindly provided by Dr. Ricardo Benavente; [76]). Histone H3 phosphorylated at serine 10 (pH3) was detected with a polyclonal rabbit serum (Upstate, Lake Placid, New York, United States) at a 1:1,000 dilution. Following three washes in PBS for 5 minutes, the slides were incubated for 30 minutes at room temperature with secondary antibodies. A fluorescein isothiocyanate (FITC)-conjugated goat anti-rabbit IgG (Jackson, West Grove, Pennsylvania, United States) at a 1:150 dilution in PBS, and a Texas Red-conjugated goat anti-human IgG (Jackson) at a 1:150 dilution in PBS, were used for simultaneous double immunolabeling. The slides were subsequently rinsed in PBS, and counterstained for 3 minutes with 2 µg/ml DAPI (4′, 6-diamidino-2-phenylindole). After a final rinse in PBS, the slides were mounted in Vectashield (Vector Laboratories, Burlingame, California, United States) and sealed with nail varnish. In double immunolabeling experiments, primary antibodies were incubated simultaneously except when primary antibodies were generated in the same host species when we proceeded as previously described ([77]). In those cases slides were first incubated with the rabbit serum against hCAP-H for 1 hour at room temperature, rinsed in PBS and incubated overnight at 4°C with an FITC-conjugated goat Fab' fragment anti-rabbit IgG (Jackson) at a 1:100 dilution in PBS. Afterwards, slides were rinsed six times for 5 minutes in PBS, incubated with the rabbit antibody against TRF1 or RAD21 for 1 hour, rinsed three times for 5 minutes in PBS, and incubated with a Texas Red-conjugated goat anti-rabbit IgG (Jackson) at a 1:150 dilution.

Observations were performed using an Olympus BH-2 microscope (Olympus, Hamburg, Germany) equipped with epifluorescence optics and the images were recorded with an Olympus DP50 digital camera. Digital images were then treated using the Adobe PhotoShop 7.0 software.

## Supporting Information

Figure S1 Immunoblot of HeLa cell extracts (left lane) and mouse testis extracts (right lane) probed with the anti-hCAP-H antibody. The positions of two molecular mass markers are indicated. The antibody specifically recognized a single protein band of about 90 kDa in both extracts.
(0.28 MB TIF)

Figure S2 CAP-D2 distribution in spermatogonial mitosis. Mouse spermatogonia were stained for CAP-D2 (green), kinetochores with an ACA serum (red), and counterstained with DAPI (blue). (A–C) Early prometaphase. Condensing chromosomes present a faint and diffuse CAP-D2 staining, but some bright spots are also observed. (D–F) Metaphase in top view, and (G–I) anaphase. A single diffuse CAP-D2 axis is seen in each chromosome/chromatid, as well as bright accumulations. Bar, 5 µm.
(5.93 MB TIF)

Figure S3 H3 phosphorylation precedes condensin I recruitment to chromosomes in spermatogonial mitosis. Mouse spermatogonia were stained for histone H3 phosphorylated at serine 10 (pH3) (red), CAP-H (green), and counterstained with DAPI (blue). (A–C) Middle prophase. Phosphorylated H3 is present in condensing chromosomes but not CAP-H. (D–F) Late prophase. CAP-H appears as small bright spots (arrowheads) and in the nucleoplasm. (G–I) Metaphase in top view. A single CAP-H axis (arrows) is seen in each chromosome. Bar, 5 µm.
(5.91 MB TIF)

Figure S4 Relative distributions of CAP-H and fibrillarin in prophase I spermatocytes. Mouse spermatocytes were stained for CAP-H (green), fibrillarin (red), and counterstained with DAPI (blue). (A–C) Zygotene spermatocyte. CAP-H is not detected, and fibrillarin appears at nucleoli. (D–F) Early pachytene, (G–I) late pachytene, and (J–L) diplotene spermatocytes. CAP-H and fibrillarin colocalize at the nucleoplasmic nucleoli, and at the nucleolus associated to the sex body (XY). Note that CAP-H and fibrillarin are not present at the round body and/or the fibrillar centre (arrows) inside the sex body-associated nucleolus during pachytene, and the nucleolus in diplotene (Knibiehler et al., 1981). Fibrillarin is additionally present in Cajal bodies (arrowheads) lying in the nucleoplasm (E, H) or associated to the nucleolus (K). Bar, 5 µm. Supplementary Reference: Knibiehler B, Mirre C, Hartung M, Jean P, Stahl A (1981) Sex vesicle-associated nucleolar orgnizers in mouse spermatocytes: localization, structure, and function. Cytogenet Cell Genet 31: 47–57.
(10.16 MB TIF)

Figure S5 CAP-D2 distribution in spermatocytes. Mouse spermatocytes were stained for CAP-D2 (green), kinetochores with an ACA serum (red in A–F, J–R), TRF1 (red in G–I), and counterstained with DAPI (blue). (A–C) Metaphase I spermatocyte. The autosomal bivalents show pairs of bright CAP-D2 spots at their centromeric and distal ends. (D–I) Two selected autosomal metaphase I bivalents. The proximal pair of CAP-D2 signals appears below the closely associated sister kinetochores (D–F). The four pairs of CAP-D2 signals colocalize with the TRF1 signals (G–I). (J–L) Anaphase I, (M–O) metaphase II, and (P–R) anaphase II spermatocytes. Chromosomes/chromatids show a faint CAP-D2 labeling along them, and brighter CAP-D2 dots at their ends. The insets in (J–L) show an enlarged anaphase I half-bivalent (arrows). Bars: (A–C, J–R) 5 µm; (D–I) 3 µm.
(1.14 MB JPG)

Figure S6 H3 phosphorylation at serine 10 precedes condensin I recruitment to chromosomes in meiosis I. Mouse spermatocytes were stained for histone H3 phosphorylated at serine 10 (pH3) (red), CAP-H (green), and counterstained with DAPI blue). (A–C) Diplotene spermatocyte. Phosphorylated H3 is enriched at chromocentres, while CAP-H appears at nucleoli. (D–F) Late diplotene. Phosphorylated H3 appears on all the chromatin, and CAP-H at a nucleolus. (G–I) Prometaphase I spermatocyte. CAP-H appears on bivalents, and as pairs of bright dots (double arrowheads). (J–L) Metaphase I spermatocyte. CAP-H is detected as pairs of bright spots at the centromeric and distal chromosome ends. (M–P) Two focal planes of an anaphase I spermatocyte. In each chromosome, a pair of CAP-H dots is detected at the centromere region (red arrowheads), one spot at the distal end of each chromatid (white arrowheads), and a diffuse axial labeling along chromatids. The inset shows the chromosome indicated in (O), where the CAP-H staining has been pseudocolored in red and superimposed on its corresponding DAPI image. Bar, 5 µm.
(9.96 MB TIF)

Figure S7 H3 phosphorylation precedes condensin I recruitment to chromosomes in meiosis II. Mouse spermatocytes were stained for histone H3 phosphorylated at serine 10 (pH3) (red), CAP-H (green), and counterstained with DAPI blue). (A–D) Two focal planes of a late interkinesis nucleus. Phosphorylated H3 is present on all the chromatin, whereas CAP-H appears at one nucleolus (nu) and at small spots in the nucleoplasm. (E–G) Metaphase II, and (H–K) two focal planes of an anaphase II spermatocyte. Bright CAP-H spots are present at chromosome ends. Bar, 5 µm.
(5.26 MB TIF)

Figure S8 Relative distributions of CAP-H and TRF1 in spermatogonial metaphase chromosomes. (A–D) Mouse metaphase spermatogonia stained for CAP-H (green), TRF1 (red), and counterstained with DAPI (blue). A fuzzy CAP-H axis is visible inside each sister chromatid. The bright CAP-H accumulations at the axes ends colocalize with TRF1. Bar, 5 µm.
(2.25 MB TIF)

Figure S9 Silver staining of metaphase I spermatocytes. (A) Metaphase I spread spermatocyte where the sex bivalent is indicated (XY), and selected autosomal bivalents (B, D) and sex (C) bivalent. Two silver-stained structures representing sister kinetochores (arrowheads) are found at the centromeric region of each homologue. Faint silver-stained axes (arrows) are observed along the inner region of the chromatids. (E) Autosomal metaphase I bivalent shown in Fig. 2N stained for CAP-H (pseudocolored in red), and counterstained with DAPI (blue). The CAP-H axes are located inside the chromatids as the silver-stained chromatid axes (D). Bars: (A) 10 µm; (B–E) 3 µm.
(5.41 MB TIF)
