# Supplementary figures and images for: Condensin I Reveals New Insights on Mouse Meiotic Chromosome Structure and Dynamics
Source: PLoS One. 2007 Aug 22;2(8):e783. doi: 10.1371/journal.pone.0000783 (PMC1942118; doi:10.1371/journal.pone.0000783)

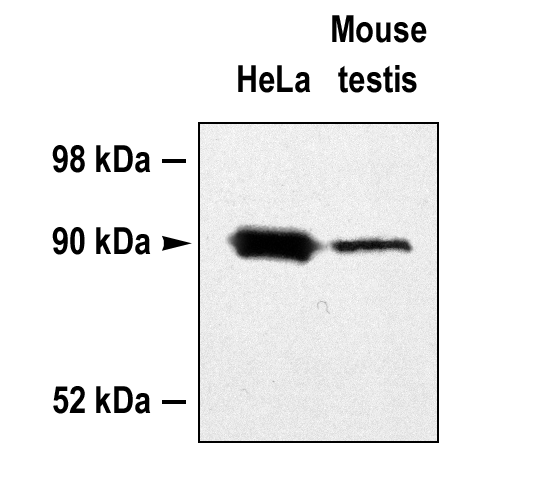

Supplement: Figure S1 — Immunoblot of HeLa cell extracts (left lane) and mouse testis extracts (right lane) probed with the anti-hCAP-H antibody. The positions of two molecular mass markers are indicated. The antibody specifically recognized a single protein band of about 90 kDa in both extracts. (0.28 MB TIF) [file pone.0000783.s001.tif]

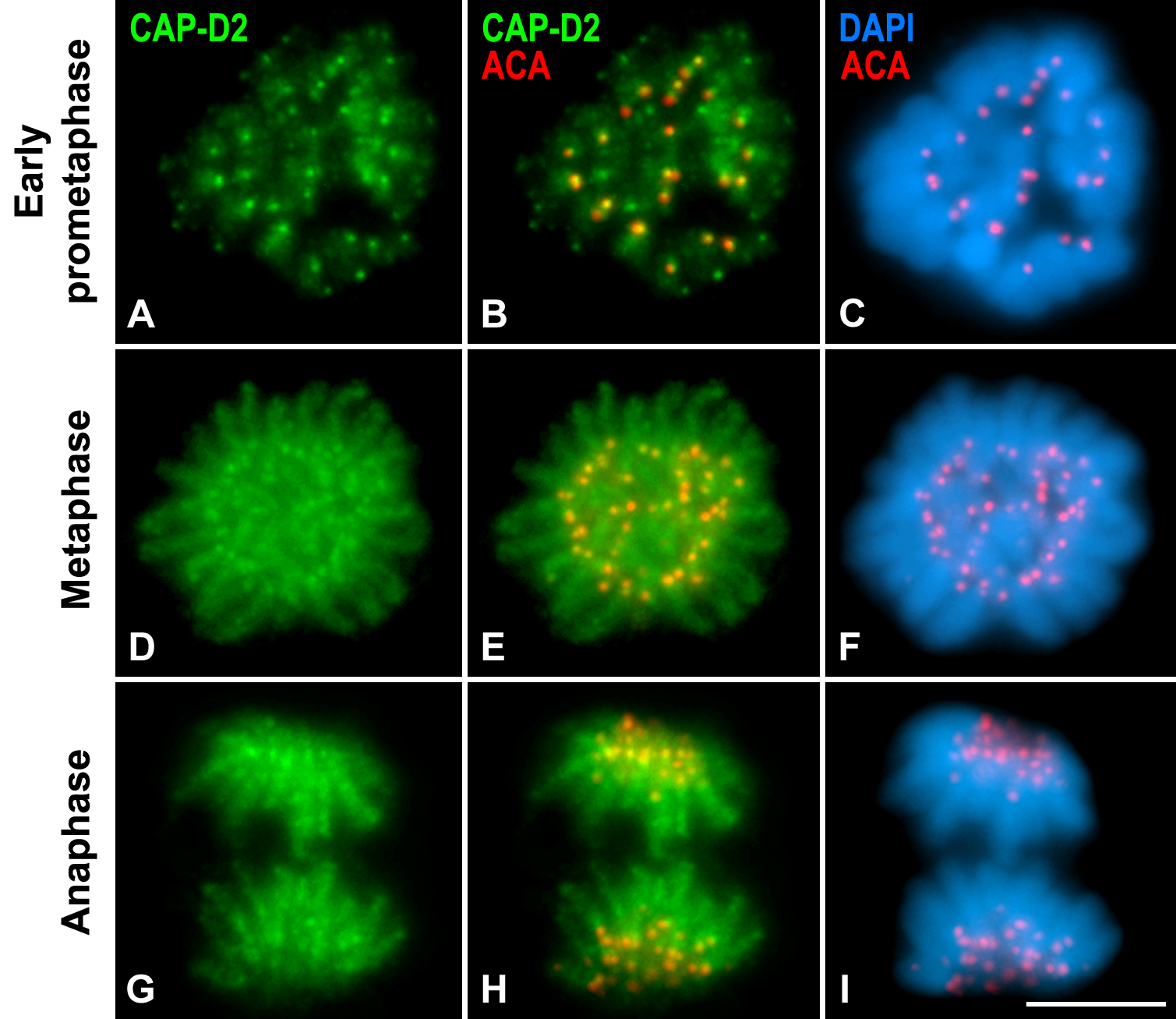

Supplement: Figure S2 — CAP-D2 distribution in spermatogonial mitosis. Mouse spermatogonia were stained for CAP-D2 (green), kinetochores with an ACA serum (red), and counterstained with DAPI (blue). (A–C) Early prometaphase. Condensing chromosomes present a faint and diffuse CAP-D2 staining, but some bright spots are also observed. (D–F) Metaphase in top view, and (G–I) anaphase. A single diffuse CAP-D2 axis is seen in each chromosome/chromatid, as well as bright accumulations. Bar, 5 µm. (5.93 MB TIF) [file pone.0000783.s002.tif]

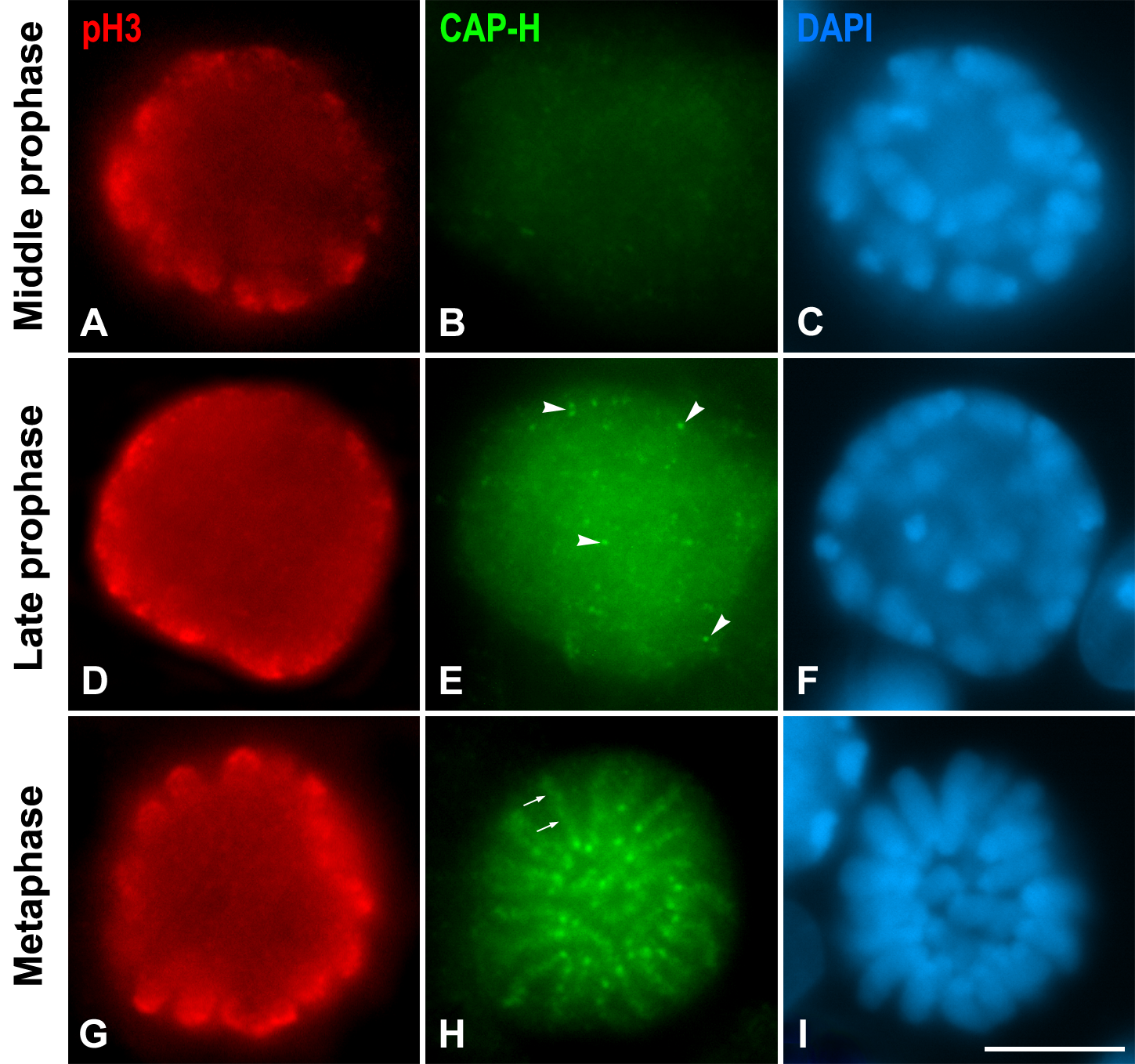

Supplement: Figure S3 — H3 phosphorylation precedes condensin I recruitment to chromosomes in spermatogonial mitosis. Mouse spermatogonia were stained for histone H3 phosphorylated at serine 10 (pH3) (red), CAP-H (green), and counterstained with DAPI (blue). (A–C) Middle prophase. Phosphorylated H3 is present in condensing chromosomes but not CAP-H. (D–F) Late prophase. CAP-H appears as small bright spots (arrowheads) and in the nucleoplasm. (G–I) Metaphase in top view. A single CAP-H axis (arrows) is seen in each chromosome. Bar, 5 µm. (5.91 MB TIF) [file pone.0000783.s003.tif]

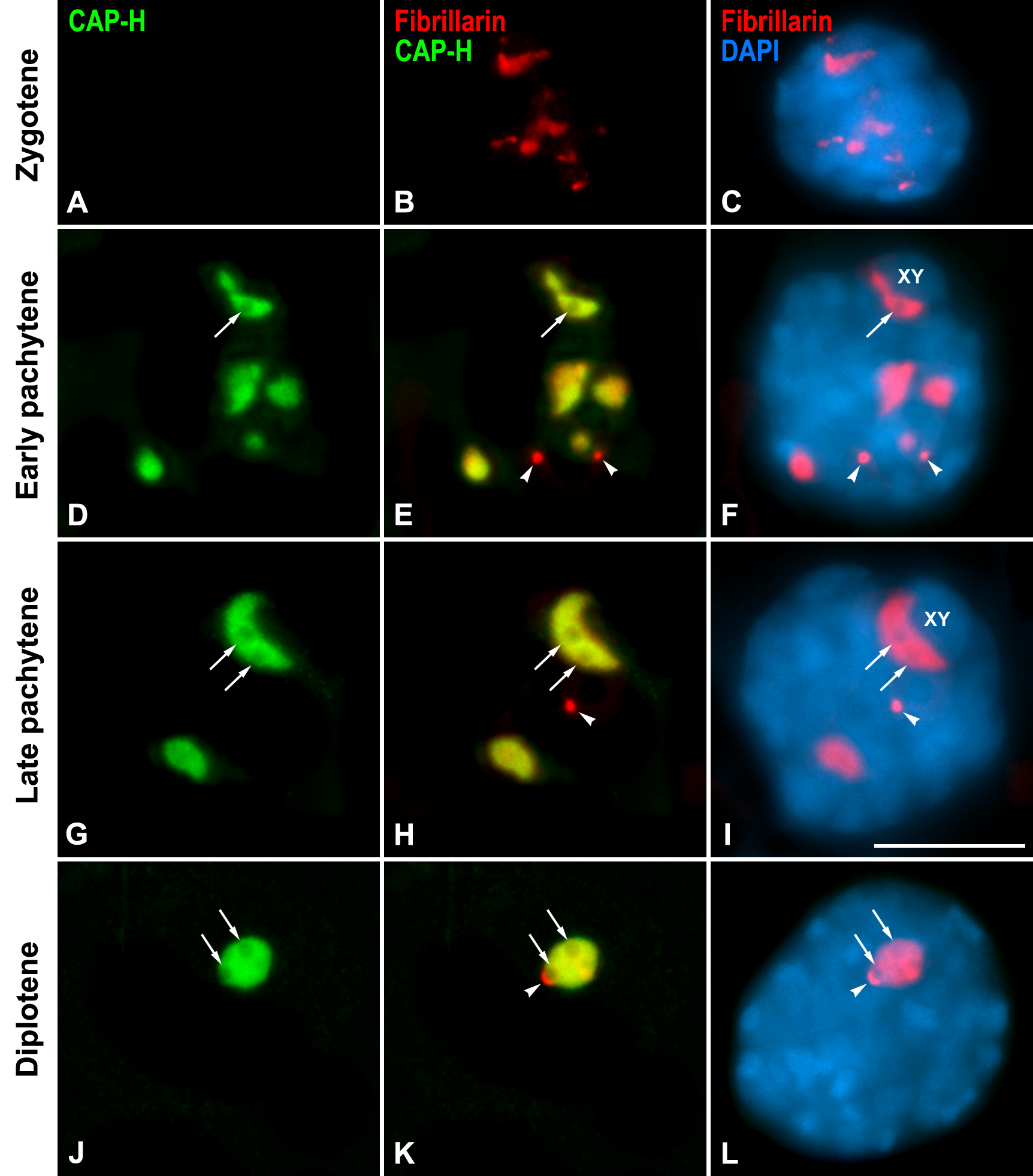

Supplement: Figure S4 — Relative distributions of CAP-H and fibrillarin in prophase I spermatocytes. Mouse spermatocytes were stained for CAP-H (green), fibrillarin (red), and counterstained with DAPI (blue). (A–C) Zygotene spermatocyte. CAP-H is not detected, and fibrillarin appears at nucleoli. (D–F) Early pachytene, (G–I) late pachytene, and (J–L) diplotene spermatocytes. CAP-H and fibrillarin colocalize at the nucleoplasmic nucleoli, and at the nucleolus associated to the sex body (XY). Note that CAP-H and fibrillarin are not present at the round body and/or the fibrillar centre (arrows) inside the sex body-associated nucleolus during pachytene, and the nucleolus in diplotene (Knibiehler et al., 1981). Fibrillarin is additionally present in Cajal bodies (arrowheads) lying in the nucleoplasm (E, H) or associated to the nucleolus (K). Bar, 5 µm. Supplementary Reference: Knibiehler B, Mirre C, Hartung M, Jean P, Stahl A (1981) Sex vesicle-associated nucleolar orgnizers in mouse spermatocytes: localization, structure, and function. Cytogenet Cell Genet 31: 47–57. (10.16 MB TIF) [file pone.0000783.s004.tif]

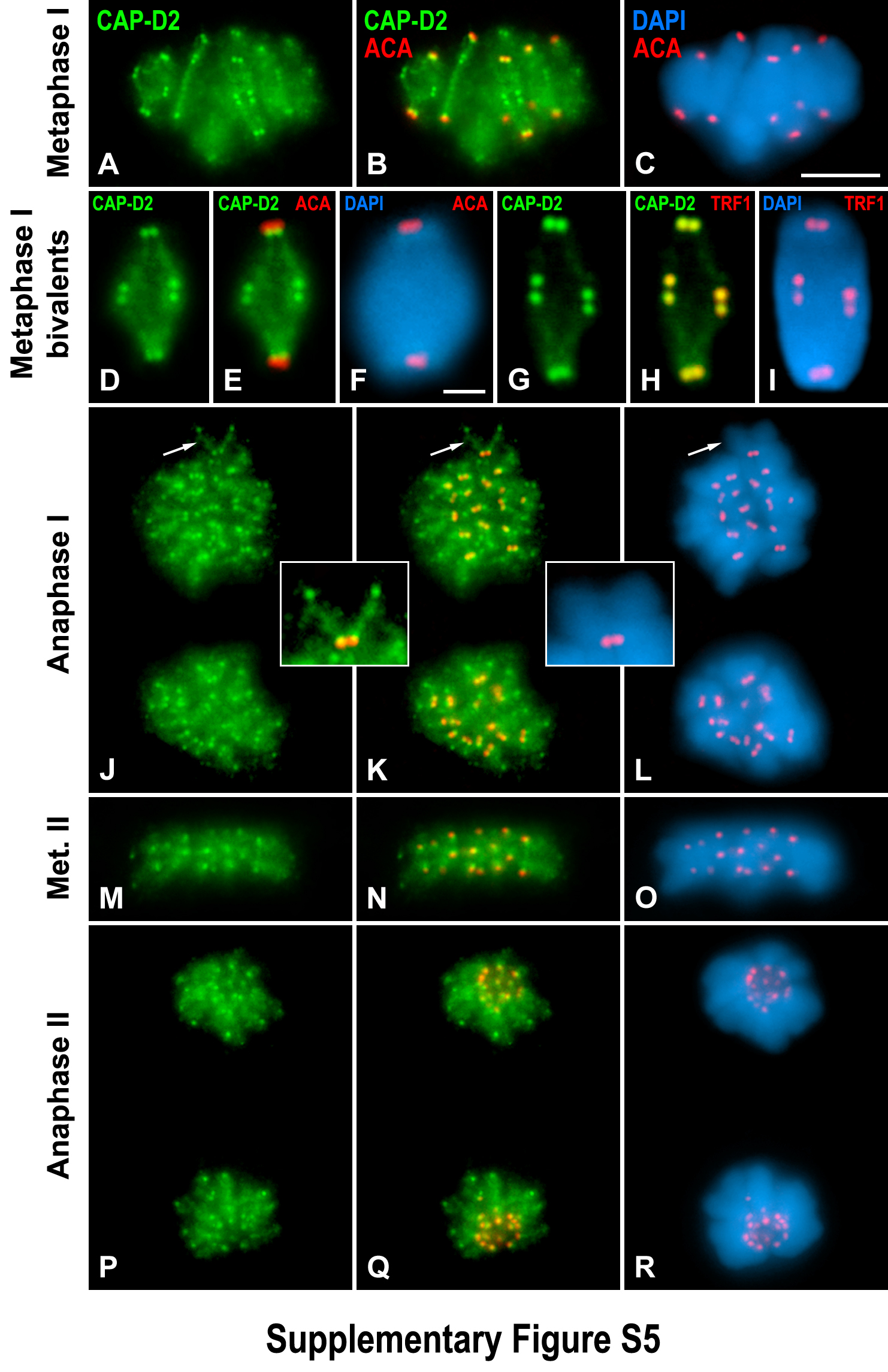

Supplement: Figure S5 — CAP-D2 distribution in spermatocytes. Mouse spermatocytes were stained for CAP-D2 (green), kinetochores with an ACA serum (red in A–F, J–R), TRF1 (red in G–I), and counterstained with DAPI (blue). (A–C) Metaphase I spermatocyte. The autosomal bivalents show pairs of bright CAP-D2 spots at their centromeric and distal ends. (D–I) Two selected autosomal metaphase I bivalents. The proximal pair of CAP-D2 signals appears below the closely associated sister kinetochores (D–F). The four pairs of CAP-D2 signals colocalize with the TRF1 signals (G–I). (J–L) Anaphase I, (M–O) metaphase II, and (P–R) anaphase II spermatocytes. Chromosomes/chromatids show a faint CAP-D2 labeling along them, and brighter CAP-D2 dots at their ends. The insets in (J–L) show an enlarged anaphase I half-bivalent (arrows). Bars: (A–C, J–R) 5 µm; (D–I) 3 µm. (1.14 MB JPG) [file pone.0000783.s005.jpg]

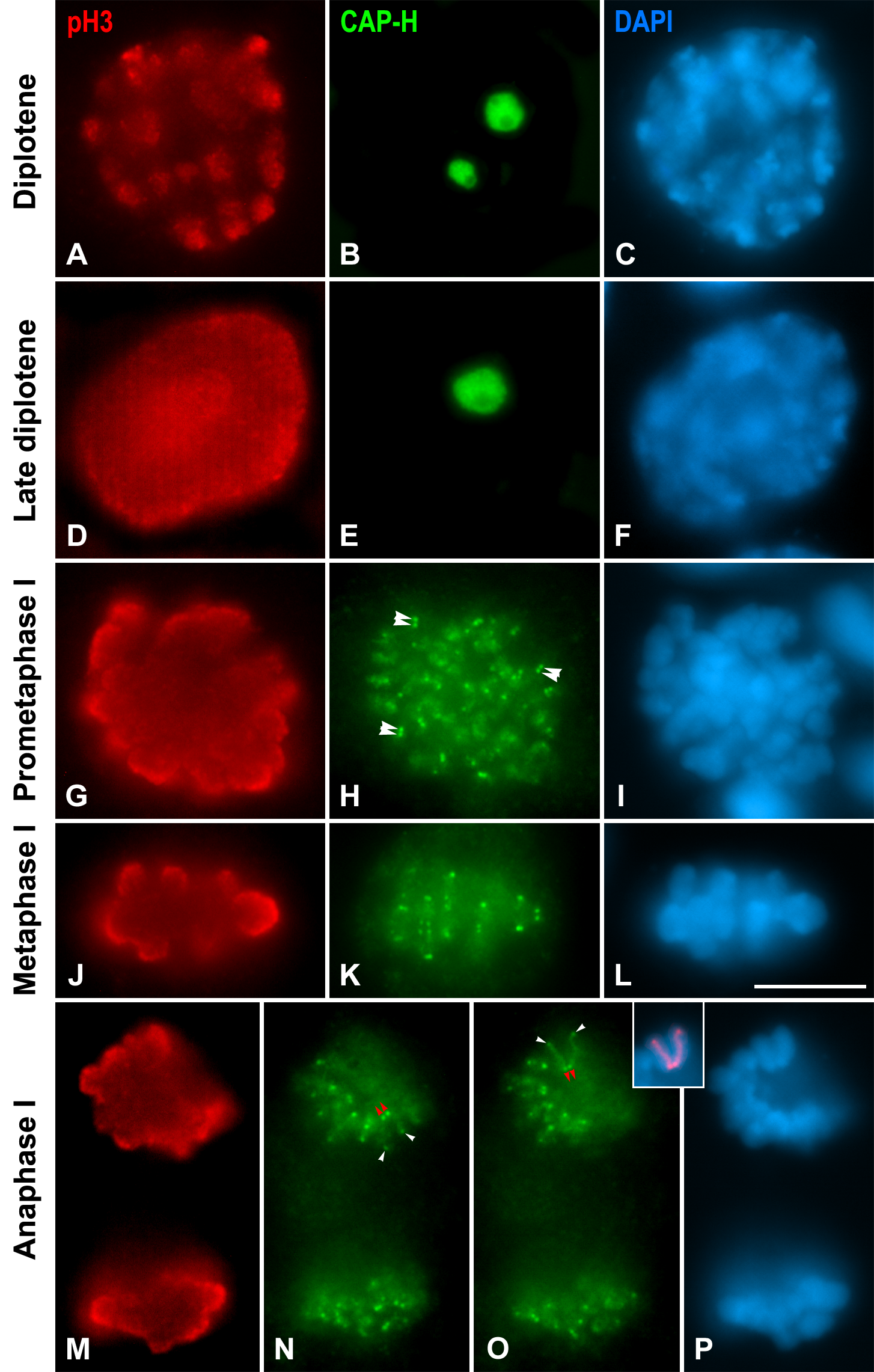

Supplement: Figure S6 — H3 phosphorylation at serine 10 precedes condensin I recruitment to chromosomes in meiosis I. Mouse spermatocytes were stained for histone H3 phosphorylated at serine 10 (pH3) (red), CAP-H (green), and counterstained with DAPI blue). (A–C) Diplotene spermatocyte. Phosphorylated H3 is enriched at chromocentres, while CAP-H appears at nucleoli. (D–F) Late diplotene. Phosphorylated H3 appears on all the chromatin, and CAP-H at a nucleolus. (G–I) Prometaphase I spermatocyte. CAP-H appears on bivalents, and as pairs of bright dots (double arrowheads). (J–L) Metaphase I spermatocyte. CAP-H is detected as pairs of bright spots at the centromeric and distal chromosome ends. (M–P) Two focal planes of an anaphase I spermatocyte. In each chromosome, a pair of CAP-H dots is detected at the centromere region (red arrowheads), one spot at the distal end of each chromatid (white arrowheads), and a diffuse axial labeling along chromatids. The inset shows the chromosome indicated in (O), where the CAP-H staining has been pseudocolored in red and superimposed on its corresponding DAPI image. Bar, 5 µm. (9.96 MB TIF) [file pone.0000783.s006.tif]

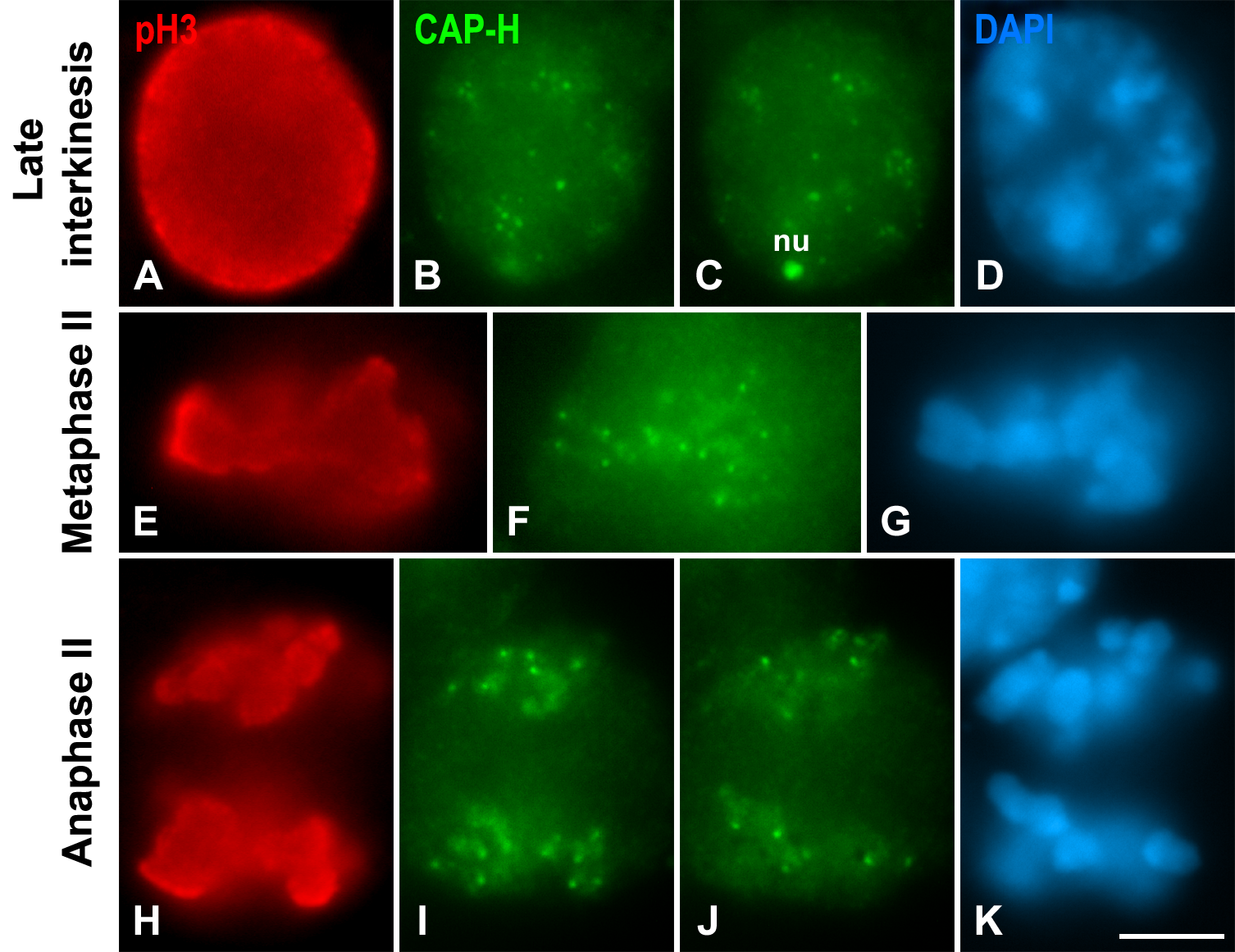

Supplement: Figure S7 — H3 phosphorylation precedes condensin I recruitment to chromosomes in meiosis II. Mouse spermatocytes were stained for histone H3 phosphorylated at serine 10 (pH3) (red), CAP-H (green), and counterstained with DAPI blue). (A–D) Two focal planes of a late interkinesis nucleus. Phosphorylated H3 is present on all the chromatin, whereas CAP-H appears at one nucleolus (nu) and at small spots in the nucleoplasm. (E–G) Metaphase II, and (H–K) two focal planes of an anaphase II spermatocyte. Bright CAP-H spots are present at chromosome ends. Bar, 5 µm. (5.26 MB TIF) [file pone.0000783.s007.tif]

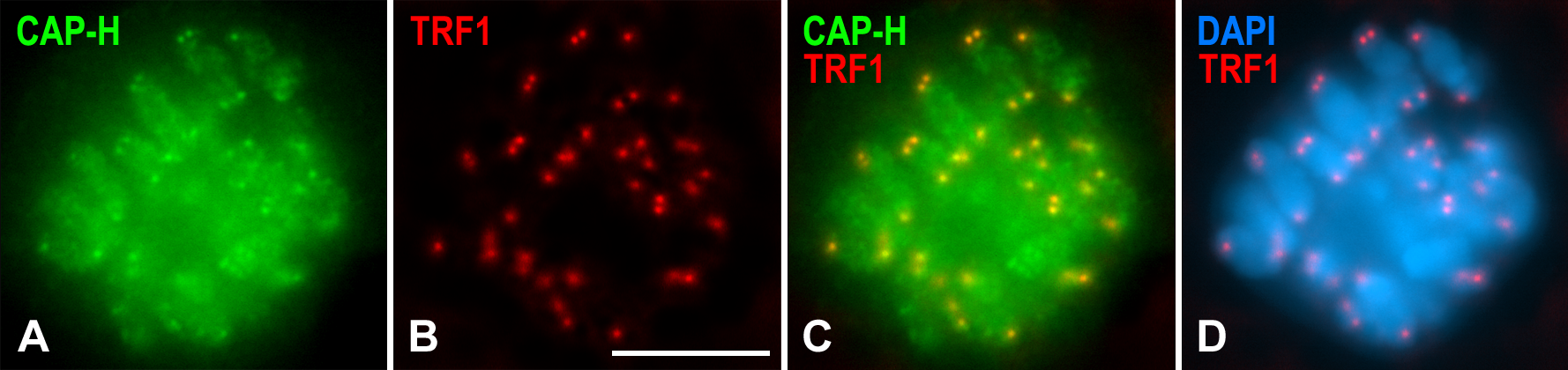

Supplement: Figure S8 — Relative distributions of CAP-H and TRF1 in spermatogonial metaphase chromosomes. (A–D) Mouse metaphase spermatogonia stained for CAP-H (green), TRF1 (red), and counterstained with DAPI (blue). A fuzzy CAP-H axis is visible inside each sister chromatid. The bright CAP-H accumulations at the axes ends colocalize with TRF1. Bar, 5 µm. (2.25 MB TIF) [file pone.0000783.s008.tif]

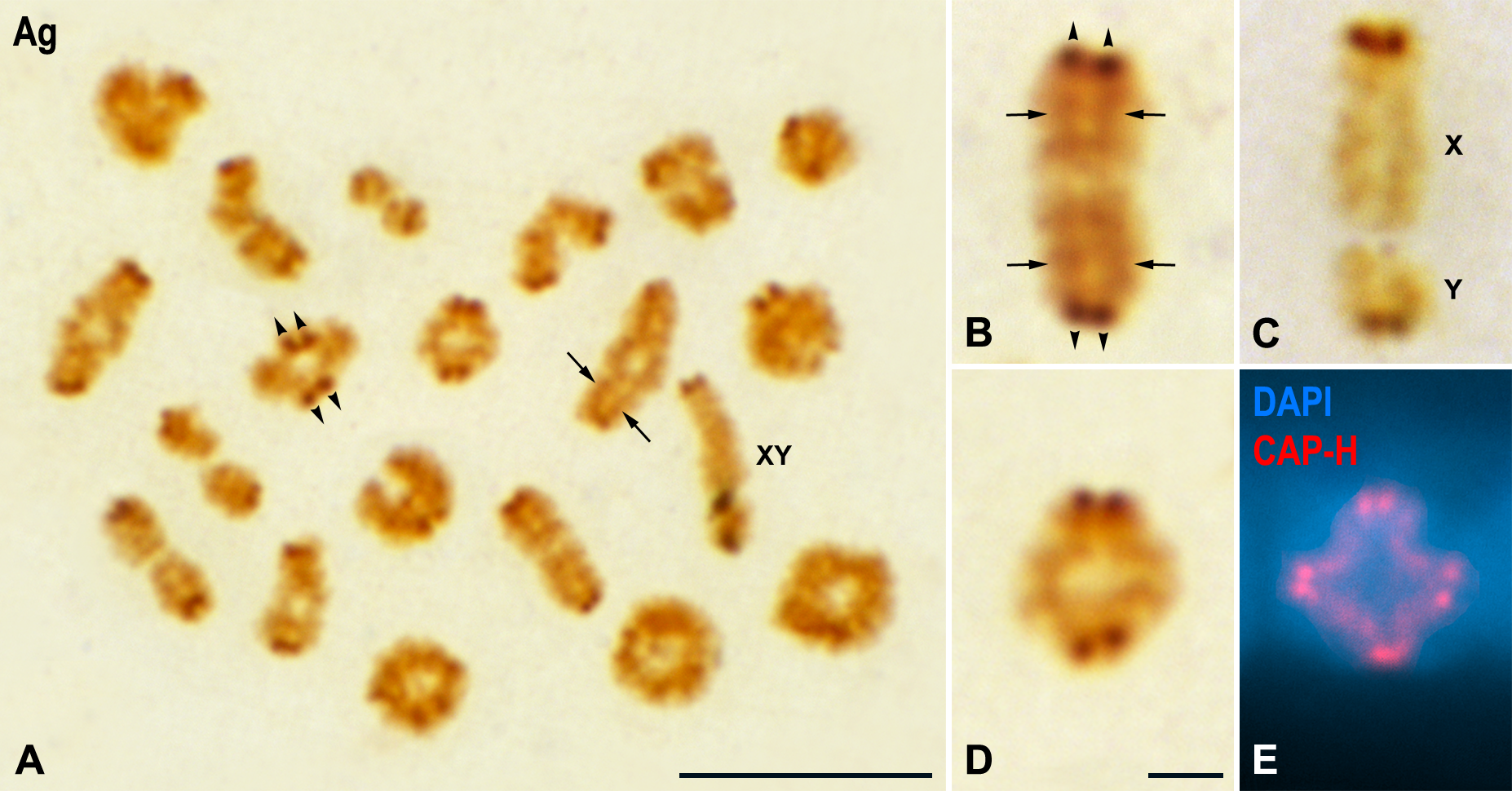

Supplement: Figure S9 — Silver staining of metaphase I spermatocytes. (A) Metaphase I spread spermatocyte where the sex bivalent is indicated (XY), and selected autosomal bivalents (B, D) and sex (C) bivalent. Two silver-stained structures representing sister kinetochores (arrowheads) are found at the centromeric region of each homologue. Faint silver-stained axes (arrows) are observed along the inner region of the chromatids. (E) Autosomal metaphase I bivalent shown in Fig. 2N stained for CAP-H (pseudocolored in red), and counterstained with DAPI (blue). The CAP-H axes are located inside the chromatids as the silver-stained chromatid axes (D). Bars: (A) 10 µm; (B–E) 3 µm. (5.41 MB TIF) [file pone.0000783.s009.tif]
